# Expression, purification, crystallization and preliminary crystallographic analysis of a putative *Clostridium difficile* surface protein Cwp19

**DOI:** 10.1107/S1744309111016770

**Published:** 2011-06-30

**Authors:** Jonathan M. Kirby, Nethaji Thiyagarajan, April K. Roberts, Clifford C. Shone, K. Ravi Acharya

**Affiliations:** a Department of Biology and Biochemistry, University of Bath, Claverton Down, Bath BA2 7AY, England; b Research Department, Health Protection Agency, Porton Down, Salisbury SP4 0JG, England

**Keywords:** Cwp19, *Clostridium difficile*, surface proteins

## Abstract

Cwp19 is a putatively surface-located protein from *Clostridium difficile*. A recombinant N-terminal protein (residues 27–401) lacking the signal peptide and the C-terminal cell-wall-binding repeats (PFam04122) was crystallized using the sitting-drop vapour-diffusion method and diffracted to 2 Å resolution. The crystal appeared to belong to the primitive monoclinic space group *P*2_1_, with unit-cell parameters *a* = 109.1, *b* = 61.2, *c* = 109.2 Å, β = 111.85°, and is estimated to contain two molecules of Cwp19 per asymmetric unit.

## Abstract 2

Cwp19 is a putatively surface-located protein from *Clostridium difficile*. A recombinant N-terminal protein (residues 27–401) lacking the signal peptide and the C-terminal cell-wall-binding repeats (PFam04122) was crystallized using the sitting-drop vapour-diffusion method and diffracted to 2 Å resolution.

## 1. Introduction

*Clostridium difficile* is a Gram-positive spore-forming primarily nosocomial pathogen that is the aetiological agent in antibiotic-associated diarrhoea and pseudomembranous colitis (Bartlett, 2010 ▶). Changes in epidemiology and disease severity, particularly in strains that have emerged over the last ten years, *e.g.* the 027 ribotype, highlight the need to understand more about this worldwide pathogen (Freeman *et al.*, 2010 ▶).

The elucidation of structural information for *C. difficile* proteins has understandably been directed towards the main virulence factors, the toxins (Albesa-Jové *et al.*, 2010 ▶; Ho *et al.*, 2005 ▶; Pruitt *et al.*, 2009 ▶, 2010 ▶; Sundriyal *et al.*, 2009 ▶). Despite adherence and subsequent colonization by *C. difficile* representing key milestones in infection, there are considerable gaps in the understanding of how the surface proteins of *C. difficile* interact with both themselves and the environment to mediate these key steps. To date, there is only one report of high-resolution structural information for a *C. difficile* surface protein: the low-molecular-weight subunit of the S-layer (PDB entry 3cvz; Fagan *et al.*, 2009 ▶).

The *C. difficile* S-layer is derived from post-translational cleavage of SlpA into low-molecular-weight and high-molecular-weight sub­units (LMW SLP and HMW SLP, respectively). HMW SLP contains three PFam04122 repeats which putatively mediate attachment to the bacterial cell surface (cell-wall-binding domains; CWBDs). A total of 28 other proteins in the *C. difficile* 630 genome have been found to contain these CWBDs at the N-terminus or the C-terminus, with a ‘functional domain’ at the other terminus (Sebaihia *et al.*, 2006 ▶). Recently, Dang *et al.* (2010 ▶) identified one such CWBD-containing protein, Cwp19 (CD2767; *C. difficile* 630 genome numbering; Fagan *et al.*, 2011 ▶; Sebaihia *et al.*, 2006 ▶), during a pull-down assay of ABP-labelled Cwp84. Cwp19 has an N-terminal DUF187 domain (together with three C-­terminal CWBDs) which belongs to a glycosyl hydrolase clan of enzymes that possess a TIM barrel (a conserved protein fold consisting of eight α-helices and eight parallel β-strands that alternate along the peptide backbone, as originally identified in the conserved glycolytic enzyme triosephosphate isomerase). Other members include α-amylases and cellulases.

To understand the molecular structure of this protein, the N-­terminal domain of Cwp19, lacking the CWBDs, has been expressed, purified and crystallized for structural studies.

## 2. Materials and methods

### 2.1. Cloning

A synthesized gene (GENEART, Germany) corresponding to the N-terminus lacking the predicted signal peptide and CWBDs (residues 27–401) of *cwp19* from *C. difficile* QCD32g-58 was cloned into pET28a using *Nde*I and *Eco*RI. The resulting rCwp19_27–401_ protein had a 21-amino-acid leader sequence including a His_6_ tag (MGSS­HHHHHHSSGLVPRGSHM).

### 2.2. Expression and purification

The *cwp19* construct was transformed into *Escherichia coli* BL21 (DE3) Star (Invitrogen). A single colony was used to inoculate 50 ml Terrific Broth (TB) medium (Sigma) with 50 µg ml^−1^ kanamycin supplemented with 0.5% glucose and grown overnight at 303 K. The starter culture was then inoculated into 950 ml of the aforementioned supplemented TB medium and grown until the OD reached ∼0.6. Cultures were then cooled to 289 K, induced with 1 m*M* IPTG and grown for a further 16 h before harvesting by centrifugation. Cell pellets were either used directly or frozen at 253 K.

The cell pellet was thawed on ice, resuspended in immobilized metal-affinity chromatography (IMAC) binding/wash buffer (50 m*M* Tris, 0.5 *M* NaCl, 20 m*M* imidazole pH 8.0), sonicated and centrifuged to remove cell debris. IMAC was performed on an ÄKTA design FPLC (GE Healthcare) using a HisTrap HP (GE Healthcare) column equilibrated with binding/wash buffer. Elution was performed using an imidazole gradient (elution buffer: 50 m*M* Tris, 0.5 *M* NaCl, 0.5 *M* imidazole pH 8.0). Early elution peak fractions were dialysed into 50 m*M* Tris, 150 m*M* NaCl pH 8.0, 0.2 µm filtered and then concentrated in a Vivaspin-20 10k MWCO spin concentrator to approximately 167 mg ml^−1^ (as measured by the Bradford assay using 1 mg ml^−1^ BSA as the standard). Purity was assessed by SDS–PAGE and anti-His_6_ Western blot.

### 2.3. Crystallization

Using a nanodispensing robot (Art Robbins Instruments), sitting-drop vapour-diffusion crystallization trials were set up in 96-well Intelli-Plates (Art Robbins Instruments) and incubated at 289 K. Appropriate amounts of protein solution and reservoir solution were dispensed to give 2:1, 1:1 and 1:2 ratios (using 150 or 300 nl volumes). The following screens were assessed: PACT *premier*, JCSG-*plus*, Structure Screen 1 and 2 HT-96, MemGold and Morpheus (Molecular Dimensions). A large crystal appeared after ∼4 months in well D10 of Structure Screen 1 and 2 HT-96 [0.05 *M* potassium dihydrogen phosphate, 20%(*w*/*v*) PEG 8000] using a 1:1 protein:reservoir ratio.

### 2.4. X-ray data collection and processing

A total of 250 images were recorded from a single crystal of rCwp19_27–401_ using a Quantum-4 CCD detector (ADSC Systems, California, USA) with an oscillation angle of 1.0° per image, a crystal-to-detector distance of 300 mm and an exposure time of 3 s per image at 100 K (no cryoprotectant was used) on the PX beamline I04 at the Diamond Light Source (Didcot, Oxon, England). The diffraction data were processed using the *iMOSFLM* X-ray data-processing package (Battye *et al.*, 2011 ▶) and were scaled using *SCALA* (part of the *CCP*4 program suite; Winn *et al.*, 2011 ▶). Data-collection and processing statistics are listed in Table 1 ▶. Molecular-replacement trials were attempted using the *PHENIX* suite of crystallography programs (Adams *et al.*, 2010 ▶).

## 3. Results and discussion

### 3.1. Protein expression and crystallization

Despite the identification of 28 SlpA paralogues containing a Pfam 04122 (cell-wall-binding domain, CWBD), only 11 have been either identified on the cell surface or have had their transcription demonstrated (Calabi *et al.*, 2001 ▶; Karjalainen *et al.*, 2001 ▶; Wright *et al.*, 2005 ▶). The role of CWBD-containing surface proteins in the physiology and pathogenesis of *C. difficile* has therefore only started to be understood and requires further work.

**Figure 1:**
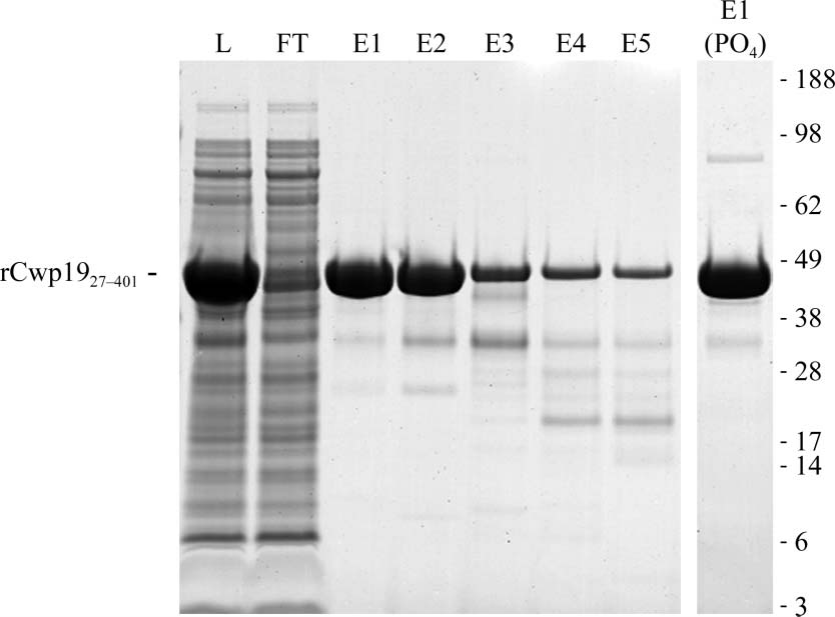
Purification of rCwp19_27–401._ The first seven lanes contain material obtained using Tris-based IMAC buffers. Lane L, *E. coli* lysate. Lane FT, unbound material. Lanes E1–5, eluted fractions from early (E1) and late (E5) in the eluted peak. Lane E1 (PO_4_), early-eluted fraction from sodium phosphate (monobasic) based IMAC buffers.

To obtain pure rCwp19 it was necessary to express only the N-­terminal functional domain, residues 27–401 (minus the predicted signal peptide, residues 1–26), containing the predicted glycosidase catalytic core. The full-length protein (including the CWBDs but also lacking the signal peptide) exhibited extensive truncation/degradation and purification issues. IMAC purification yielded a pure (>90%) 47 kDa species in one step, particularly early in the elution peak (Fig. 1 ▶). rCwp19_27–401_ had a tendency to dimerize when purified or dialysed in phosphate buffers. However, we could concentrate the protein to a final concentration of 167 mg ml^−1^.

**Figure 2:**
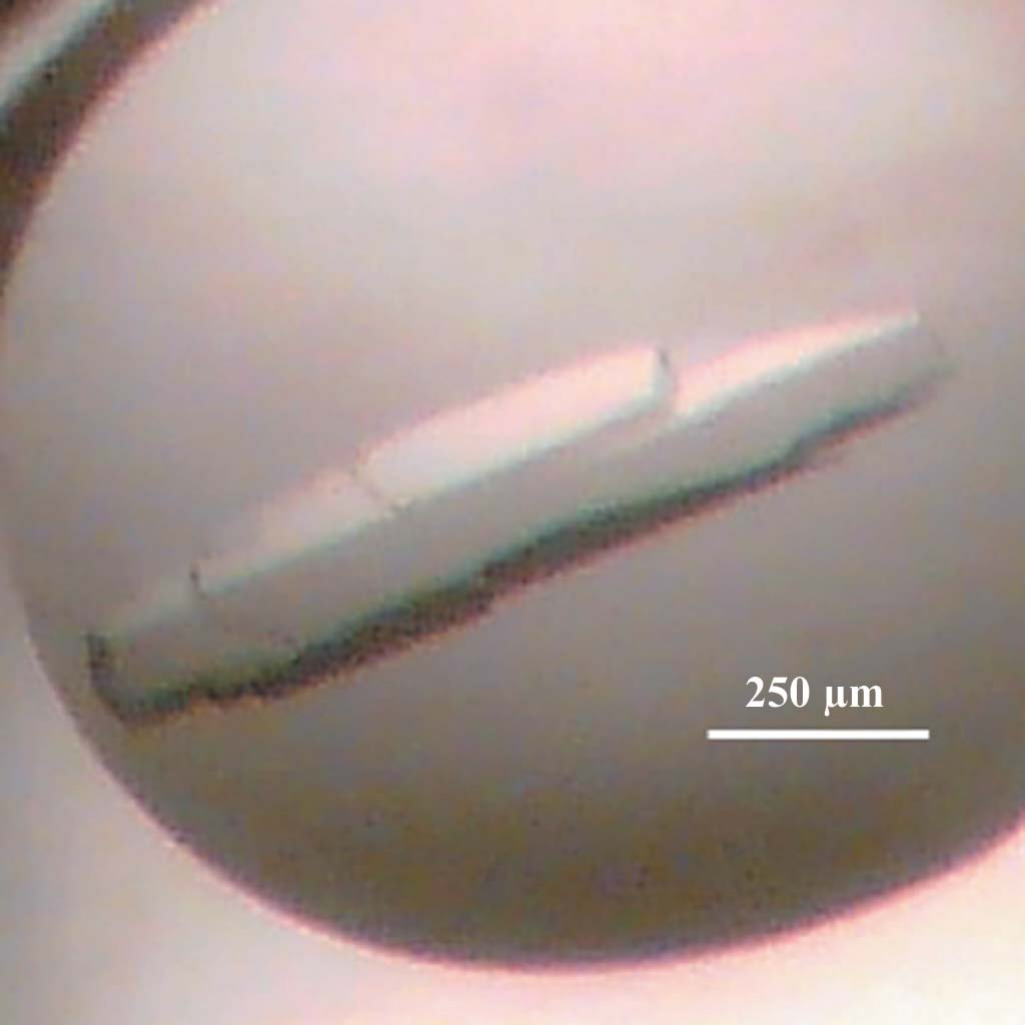
Crystal of rCwp19_27–401._

**Figure 3:**
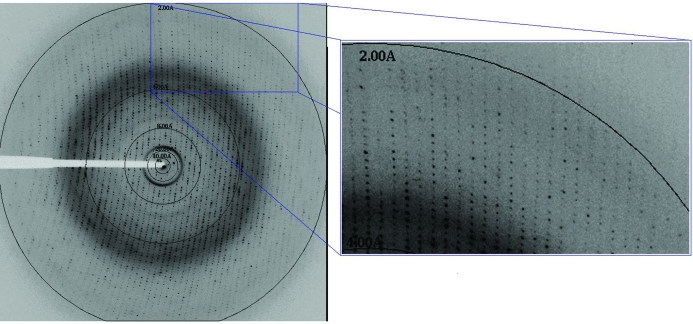
X-ray diffraction image collected from the crystal of rCwp19_27–401_ at Diamond Light Source (Oxon, England).

Using an automated high-throughput sitting-drop vapour-diffusion technique, crystals were obtained in condition D10 of Structure Screen 1 and 2 HT-96 [0.05 *M* potassium dihydrogen phosphate, 20%(*w*/*v*) PEG 8000]. The crystal (Fig. 2 ▶) grew after approximately four months and diffracted to 2.0 Å resolution (Fig. 3 ▶).

### 3.2. Space-group ambiguity

The X-ray diffraction data for the crystal of rCwp19_27–401_ were analyzed by processing the data in all suggested space groups using the *iMOSFLM* software suite (Battye *et al.*, 2011 ▶). The data were processed in centred orthorhombic, centred and primitive monoclinic and primitive triclinic space groups. The final data-processing statistics for all of these possible space groups are given in Table 1 ▶. *POINTLESS* (Winn *et al.*, 2011 ▶) suggested the primitive monoclinic system as a possible space group for the rCwp19_27–401_ crystal; how­ever, we also analysed the data for the presence of pseudotranslational symmetry (Adams *et al.*, 2010 ▶; Winn *et al.*, 2011 ▶; Vagin & Teplyakov, 1997 ▶; Vaguine *et al.*, 1999 ▶) and complete/partial mero­hedral twinning (Padilla & Yeates, 2003 ▶; French & Wilson, 1978 ▶; Adams *et al.*, 2010 ▶; Winn *et al.*, 2011 ▶). These analyses were performed for data processed in centred orthorhombic, primitive monoclinic and primitive triclinic space groups using *TRUNCATE* (Winn *et al.*, 2011 ▶; French & Wilson, 1978 ▶), *phenix.xtriage* (Adams *et al.*, 2010 ▶), the *L*-­test (Adams *et al.*, 2010 ▶; Padilla & Yeates, 2003 ▶) and the *H*-test (Lebedev *et al.*, 2006 ▶). Patterson maps were calculated using *MOLREP* (Vagin & Teplyakov, 2010 ▶) and *POLARRFN* from the *CCP*4 package (Winn *et al.*, 2011 ▶).

#### 3.2.1. Twinning analysis

*TRUNCATE* analysis showed normalized structure amplitudes 〈*E*〉 of 0.928 and 0.889 for the centred orthorhombic and primitive monoclinic space groups, respectively. The expected value for an untwinned data set is 0.886 and that for a perfectly twinned data set is 0.94. Thus, *TRUNCATE* indicated the presence of partial twinning in the centred orthorhombic space group with a twin fraction of 0.218. Twinning was not detected by *TRUNCATE* in the primitive monoclinic space group.

**Figure 4:**
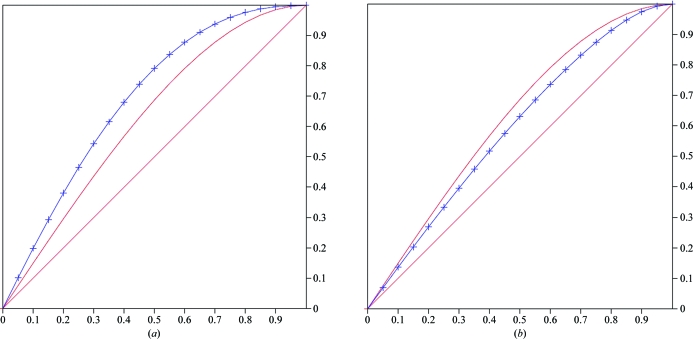
*L*-test analysis for space groups *C*222/*C*222_1_ (*a*) and *P*2/*P*2_1_ (*b*). Curved line, perfect twin; straight line, untwinned; blue line with marks, observed data.

The *L*-test analysis (Adams *et al.*, 2010 ▶; Padilla & Yeates, 2003 ▶) gave multivariate *Z* scores of 20.34 and 4.59 for the centred ortho­rhombic and primitive monoclinic space groups, respectively (Figs. 4 ▶ *a* and 4 ▶ *b*), indicating the presence of perfect twinning in the centred ortho­rhombic system. For untwinned data and where pseudosymmetry may be absent, the *Z* score is expected to be <3.5; this is not the case for the primitive monoclinic space group. The mean |*L*| values were 0.334 and 0.432 for the centred orthorhombic and primitive monoclinic systems, respectively. For a perfectly twinned case this value should be 0.375 and for an untwinned data set the value should be 0.500. In the present case, the value for the primitive monoclinic space group is closer to that for untwinned data. A similar *L*-test analysis for the primitive triclinic system resulted in a mean |*L*| value of 0.442 and a multivariate *Z* score of 3.593.

The *H*-test (Lebedev *et al.*, 2006 ▶) analysis gave a twin fraction of 0.022 for both the primitive monoclinic and primitive triclinic space groups. In the case of untwinned data the expected mean |*H*| value should be 0.50; values of 0.482 and 0.499 were found for the primitive monoclinic and primitive triclinic space groups, respectively. The *H*-­test was not performed for the centred orthorhombic system as there are no twin laws available for this space group.

The various twinning tests may appear to have erratic or high twin-fraction results because the data do not scale well in centred space groups (*C*2 or *C*222; Table 1 ▶). However, twinning may be absent in the primitive monoclinic space group.

#### 3.2.2. Pseudotranslational symmetry analysis

**Figure 5:**
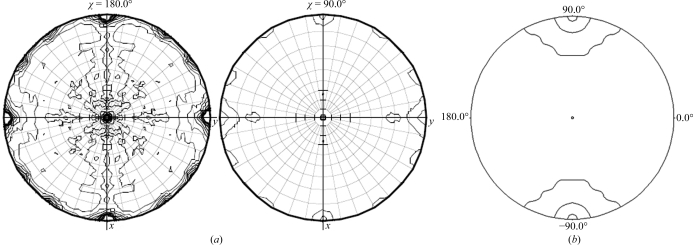
Self-rotation Patterson maps for space group *C*222 as calculated by (*a*) *MOLREP* and (*b*) *POLARRFN* (κ = 90°).

**Figure 6:**
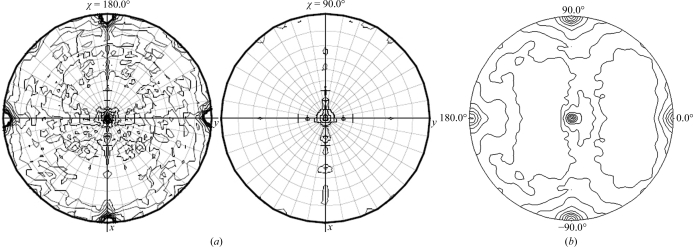
Self-rotation Patterson maps for space group *P*2 as calculated by (*a*) *MOLREP* and (*b*) *POLARRFN* (κ = 180°).

**Figure 7:**
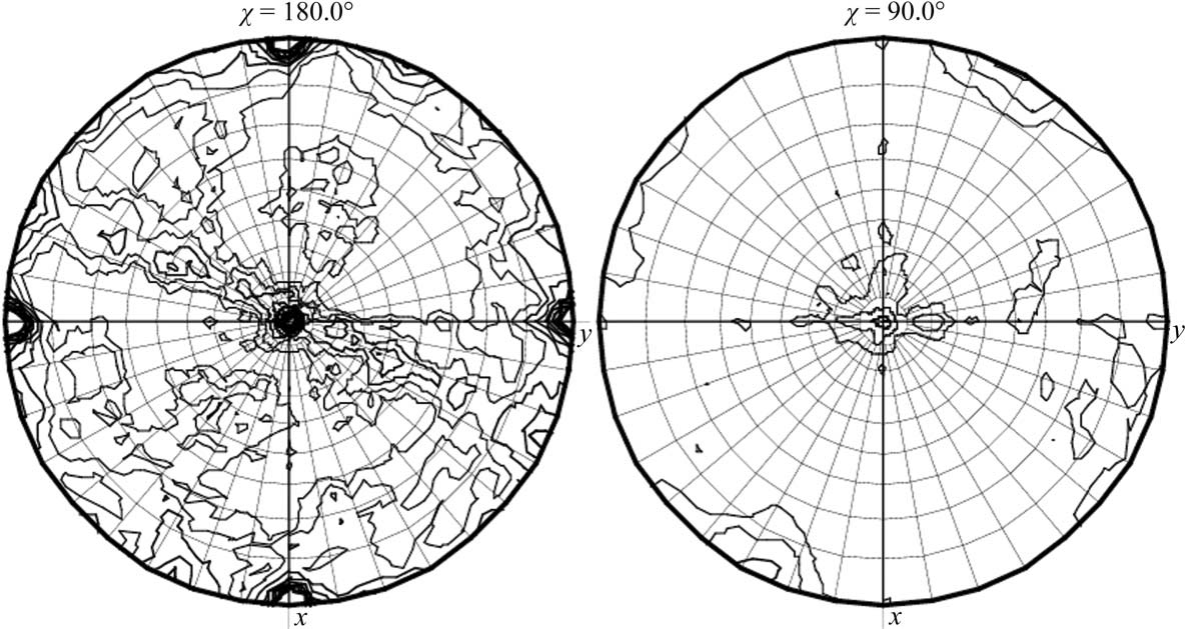
Self-rotation Patterson maps for space group *P*1 as calculated by *MOLREP*.

The presence of noncrystallographic symmetry (NCS) was tested for using *MOLREP* (Vagin & Teplyakov, 2010 ▶) and *phenix.xtriage* (Adams *et al.*, 2010 ▶). Both indicated the presence of pseudotranslational NCS in the centred orthorhombic and primitive monoclinic space groups. A strong off-origin peak was found in all these space groups. In the primitive monoclinic and primitive triclinic systems the strength of the off-origin peak was 50% of the origin peak, whereas in the centred orthorhombic space group it was only 23%. The corresponding *p*-values (calculated using *phenix.xtriage*) are 0.00520, 6.8 × 10^−5^ and 7.2 × 10^−5^ for the centred orthorhombic, primitive monoclinic and primitive triclinic systems, respectively (a *p*-value of <0.05 indicates the presence of pseudotranslational NCS). A self-rotation function was also calculated in the centred orthorhombic (Figs. 5 ▶ *a* and 5 ▶ *b*), primitive monoclinic (Figs. 6 ▶ *a* and 6 ▶ *b*) and primitive triclinic (Fig. 7 ▶) space groups using *MOLREP* and *POLARRFN* (Winn *et al.*, 2011 ▶).

#### 3.2.3. Data-processing statistics and point-group analysis

The X-­ray data-processing statistics indicated that the centred ortho­rhombic space group had an overall 〈*I*/σ(*I*)〉 of 3.6 and an overall merging *R* of 0.50, compared with the primitive monoclinic space group which had an overall 〈*I*/σ(*I*)〉 of 6.3 and an overall merging *R* of 0.135. The corresponding values for the centred monoclinic space group were 2.8 and 0.489 for the overall 〈*I*/σ(*I*)〉 and overall merging *R*, respectively. For the primitive triclinic system these values were 5.4 and 0.100 for the overall 〈*I*/σ(*I*)〉 and overall merging *R*, respectively. Similarly, the overall *R*_p.i.m._ (Evans, 2006 ▶; Leslie, 1992 ▶) values were also high for the centred orthorhombic and centred monoclinic space groups compared with the primitive monoclinic and primitive triclinic systems (Table 1 ▶).

Analysis of systematic absences (Adams *et al.*, 2010 ▶) confirmed the presence of a twofold 2_1_ screw axis in both the centred orthorhombic and primitive monoclinic space groups. There were three and two violations with 〈*I*/σ(*I*)〉 > 3.0 for the centred orthorhombic space groups *C*222 and *C*222_1_, respectively, whereas for the primitive monoclinic space groups *P*2 and *P*2_1_ there were zero and four violations with 〈*I*/σ(*I*)〉 > 3.0, respectively. However, the likelihoods for the centred orthorhombic and primitive monoclinic space groups are 7 and 1.7, respectively (as calculated using *phenix.xtriage*; Adams *et al.*, 2010 ▶).

A point-group test performed by *phenix.xtriage* (Adams *et al.*, 2010 ▶) suggested the reprocessing of data that were processed previously in the centred orthorhombic space group, which could have resulted as a consequence of over-merging of pseudo-symmetry and/or twinned data, *i.e.* this is possibly not the correct space group. A similar point-group test was carried out for data processed in  the primitive monoclinic space group, which suggested this could be the correct space group, with unit-cell parameters *a* = 109.1, *b* = 61.2, *c* = 109.2 Å, β = 111.9°. A point-group test in the primitive triclinic system also suggested a primitive monoclinic space group with identical unit-cell parameters and a likelihood score of 3.0.

Based on the various analyses performed, the data-processing statistics and suggestions from *POINTLESS* (Winn *et al.*, 2011 ▶) and *phenix.xtriage* (Adams *et al.*, 2010 ▶), we conclude that the crystal of rCwp19_27–401_ could belong to a primitive monoclinic space group. In addition, *phenix.xtriage* analysis of data processed in the primitive monoclinic space group detected the presence of pseudo-translational noncrystallographic symmetry (which could be the reason for the elevated intensity ratios observed) and twinning could be present. Hence, twin laws are applicable to this crystal symmetry and this could be the reason for the departure of the intensity statistics from normality.

### 3.3. Low sequence homology

*BLASTP* (http://blast.ncbi.nlm.nih.gov) analysis revealed that Cwp19_27–401_ has low sequence homology to known protein structures in the PDB; the closest available structure (PDB entries 1eh9 and 1eha; Feese *et al.*, 2000 ▶) shares 24% identity (44% similarity) but only over 35% of Cwp19_27–401_. Given the proposed space group, molecular-replacement trials were attempted in space group *P*2_1_ using homology models generated by *SWISS-MODEL* (Arnold *et al.*, 2006 ▶) [based on PDB entries 2gsj (Cavada *et al.*, 2006 ▶) and 3bxw (Meng *et al.*, 2010 ▶)] or *Phyre* (Kelley & Sternberg, 2009 ▶) [based on PDB entry 1m7x (Abad *et al.*, 2002 ▶)], but were unsuccessful presumably owing to low sequence identity (12.7% for 2gsj, 7.8% for 3bxw and 17% for 1m7x). Molecular modelling using the aforementioned servers together with *HHPred* (Söding *et al.*, 2005 ▶) and *I-TASSER* (Roy *et al.*, 2010 ▶) suggests that Cwp19_27–401_ has homology to proteins with a TIM-barrel structure. We are currently attempting to solve the structure of rCwp19_27–401_ using experimental phasing methods.

## Figures and Tables

**Table 1:** Statistics for the processing of X-ray data from the rCwp19_27–401_ crystal in various possible space groups using *iMOSFLM* Values in parentheses are for the highest resolution shell

Space group	*C*222/*C*222_1_	*C*2	*P*2/*P*2_1_	*P*1
Unit-cell parameters (Å, °)	*a* = 122.4, *b* = 181.18, *c* = 61.3, α = β = γ = 90.0	*a* = 122.3, *b* = 180.8, *c* = 61.2, α = γ = 90.0, β = 89.98	*a* = 109.1, *b* = 61.2, *c* = 109.2, α = γ = 90.0, β = 111.9	*a* = 61.23, *b* = 109.2, *c* = 109.3, α = 111.8, β = 90.1, γ = 89.9
Resolution range (Å)	50–2.00 (2.11–2.00)	50–2.00 (2.11–2.00)	50–2.00 (2.11–2.00)	50–2.00 (2.11–2.00)
*R*_merge_	0.502 (0.656)	0.489 (0.666)	0.135 (0.538)	0.100 (0.439)
*R*_p.i.m._	0.173 (0.258)	0.234 (0.354)	0.074 (0.306)	0.082 (0.351)
〈*I*/σ(*I*)〉	3.6 (2.2)	2.8 (1.3)	6.3 (2.6)	5.4 (2.0)
Completeness (%)	98.3 (97.8)	97.9 (95.9)	91.4 (82.9)	84.3 (75.6)
Total No. of reflections	348929 (43885)	372261 (47129)	363675 (45705)	371347 (46962)
No. of unique reflections	45719 (6575)	87734 (12542)	83202 (10948)	150187 (19737)
Multiplicity	7.6 (6.7)	4.2 (3.8)	4.4 (4.2)	2.5 (2.4)
Wilson *B* factor (Å^2^)	23.1	22.1	21.0	21.2
Average mosaicity (°)	1.2	1.1	1.1	1.1
